# Chromatin Sampling—An Emerging Perspective on Targeting Polycomb Repressor Proteins

**DOI:** 10.1371/journal.pgen.1003717

**Published:** 2013-08-22

**Authors:** Robert J. Klose, Sarah Cooper, Anca M. Farcas, Neil P. Blackledge, Neil Brockdorff

**Affiliations:** 1 Laboratory of Chromatin Biology and Transcription, Department of Biochemistry, University of Oxford, Oxford, United Kingdom; 2 Laboratory of Developmental Epigenetics, Department of Biochemistry, University of Oxford, Oxford, United Kingdom; The Babraham Institute, United Kingdom

## Overview

Polycomb group (PcG) repressor proteins play a central role in gene regulation through differentiation and development, conferring repressive chromatin configurations at target gene promoters through their inherent histone modification activities. Recruitment of Polycomb repressor proteins to defined targets has been attributed to instructive mechanisms in which sequence-specific binding proteins and/or noncoding RNAs interact biochemically with the major Polycomb repressive complexes and thus define their sites of action. Here we highlight that this viewpoint is increasingly incompatible with experimental observations. We propose an alternative perspective based on the concept that Polycomb recruitment is responsive rather than instructive. Specifically, we suggest that Polycomb complexes sample permissive chromatin sites, and through positive feedback mechanisms, accumulate at those sites lacking antagonistic chromatin modifying activities linked to ongoing transcription.

## Background

PcG repressor proteins were first discovered in *Drosophila*, where they play a specific role in maintaining the normal segmental patterns of Hox gene expression through successive cell generations. Conversely, Trithorax group (TrxG) factors were identified based on their capacity to maintain the expression of Hox gene loci. Subsequent studies revealed that both PcG and TrxG proteins are highly conserved in multicellular organisms, where they perform an essential and pervasive role in epigenetic regulation of gene expression in differentiation and development [1]. Early studies focused on the capacity of PcG factors to establish stable heritable silencing at target gene promoters [2],[3]. However, more recent genome-wide studies have revealed that PcG silencing is more dynamic than previously appreciated [4]. This is most apparent in mammalian cells where the identity of PcG factor–associated gene promoters often varies significantly between specific cell types [5]–[7]. Similarly, during X chromosome inactivation in mammals, recruitment of PcG factors is reversible, being dependent on continuous expression of the ncRNA Xist [8].

**Figure 1:**
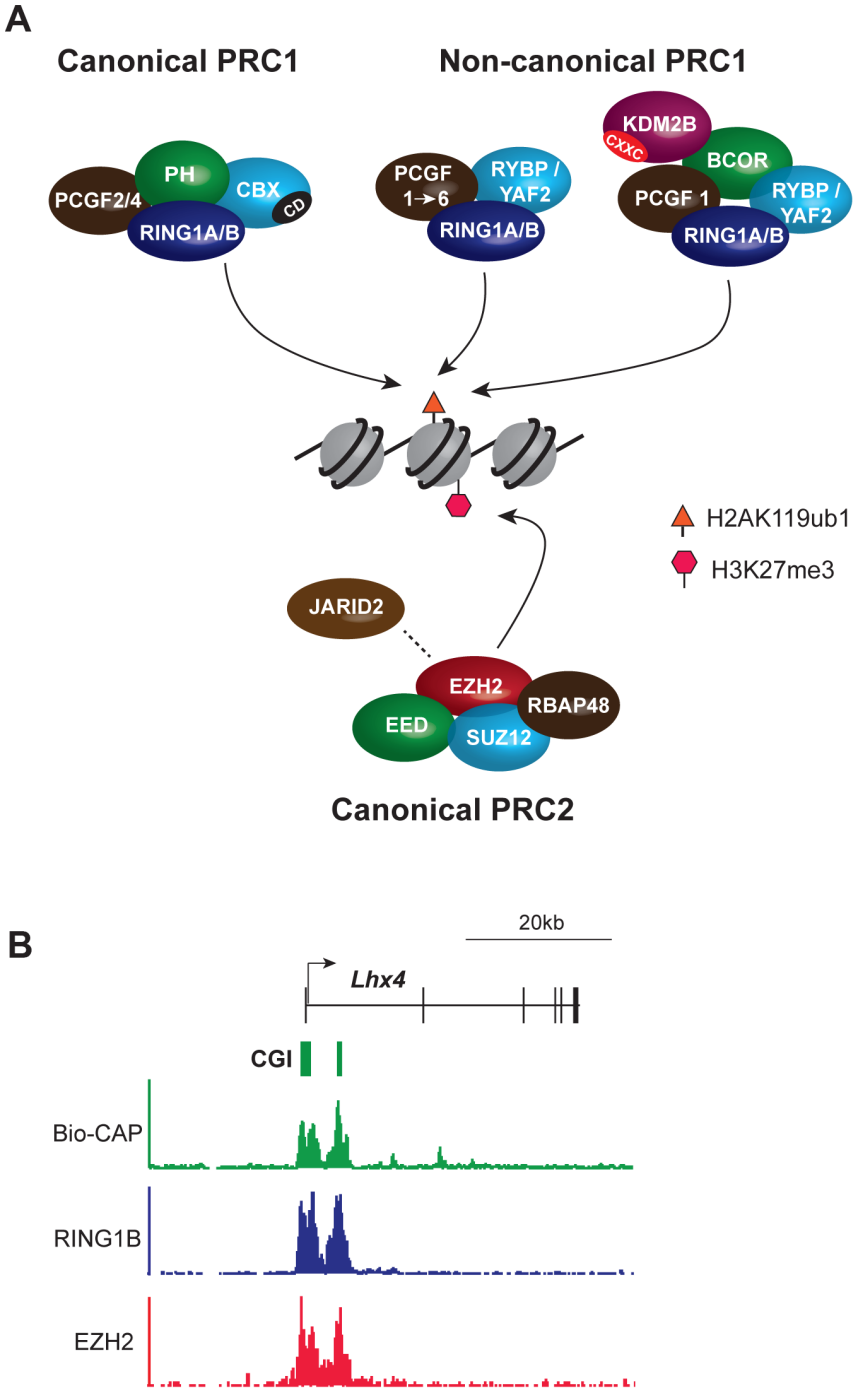
Core components of the major PcG complexes in vertebrate cells overlap with CpG islands. (A) PRC1 complexes catalyze monoubiquitylation of histone H2A lysine 119 (H2AK119ub1). The catalytic subunits are RING1A/B and one of six PCGF protein homologues, PCGF1–6. PRC1 exists in canonical and noncanonical forms. Canonical PRC1 complexes comprise RING1A/B, PCGF2/4, the CBX protein subunit that has a chromodomain (CD) that binds specifically to PRC2-mediated H3K27me3, and the PH subunit. In noncanonical PRC1, CBX proteins are substituted by the RYBP/YAF2 subunit and the PH subunit is absent. Association of additional subunits with noncanonical PRC1 occurs in a manner dependent on the associated PCGF protein. Thus, PCGF1 complexes, discussed extensively herein, include the additional subunits BCOR and KDM2B. KDM2B has a zinc finger CxxC domain (CXXC) that mediates binding to unmethylated CpG dinucleotides. PRC2 complexes methylate histone H3 lysine 27 (H3K27me1/2/3) and comprise the catalytic EZH2 protein and the core subunits EED, SUZ12, and RBAP48. JARID2 is a substoichiometric subunit that has been implicated in PRC2 targeting. (B) PcG complexes occupy CpG islands at target gene promoters: an example from genome-wide ChIP-seq analysis of mouse embryonic stem cells illustrating a PcG target gene, Lhx4. The Bio-CAP procedure provides a molecular readout of the density of unmethylated CpG dinucleotides with peaks corresponding to CpG islands. ChIP-seq for PRC1 (RING1B) and PRC2 (EZH2) subunits illustrates that PcG protein occupancy closely mirrors unmethylated CpG density.

Biochemical and genetic studies have revealed that PcG proteins generally associate with one of two multi-subunit chromatin modifying complexes, called Polycomb repressive complex (PRC) 1 and 2 (reviewed in [9]) (Figure 1A). These complexes catalyze defined histone tail modifications, with PRC1 mono-ubiquitylating histone H2A (H2AK119ub1) and PRC2 methylating histone H3 lysine 27 (H3K27me3) (Figure 1A). In vertebrates, PRC1-related complexes subdivide into canonical forms in which the catalytic subunits are associated with a homologue of the *Drosophila* proteins Polycomb (CBX2/4/6/7/8) and Polyhomeotic (PH1/2/3), and noncanonical forms in which catalytic subunits interact with one of two closely related proteins RYBP or YAF2 (Figure 1 and [10], [11]). Canonical PRC1 complexes mediate crosstalk with PRC2 complexes via interaction of a chromodomain in the CBX proteins with PRC2-mediated H3K27me3 [12], [13].

TrxG proteins similarly form multiprotein chromatin modifying complexes [14]. These include the SWI-SNF and NURF histone remodelling complexes and two separate histone methyltransferase complexes that deposit H3 lysine 4 (H3K4me3) or H3 lysine 36 (H3K36me2/3). H3K4me3 is deposited at active gene promoters and H3K36me3 over the body of transcribed loci. The catalytic activities of PcG and TrxG complexes underpin their effector functions by affecting directly, or indirectly, engagement of the transcriptional machinery.

## Instructive Models for PcG Complex Targeting

The recruitment of TrxG complexes has been linked to binding of sequence-specific transcription factors (TFs) and establishment of transcription complexes. Similarly, studies examining the *Drosophila* Hox loci have identified promoter-linked elements, PREs (polycomb response elements), that function as landing platforms for sequence-specific binding factors that are thought to directly recruit PcG complexes [3], [15]–[17]. Building on this, it has been proposed that PcG recruitment to Hox loci in *Drosophila* occurs in a stepwise process with binding of the sequence-specific TFs, notably Pho, resulting in direct recruitment of PRC2 complexes. In turn, H3K27me3 deposited on histones by PRC2 is thought to result in the hierarchical recruitment of canonical PRC1 through its intrinsic capacity to recognize this modification [18]. This model has been extrapolated to vertebrate systems where sequence-specific binding factors, for example YY1, the direct homologue of Pho [19], REST [20]–[22], and Runx1 [23], have all been suggested to recruit PcG complexes via direct biochemical interactions. However, these individual examples of transcription factor–specific targeting fail to account for most PcG occupied sites in vivo, with genome-wide analysis instead indicating that PcG protein occupancy correlates most precisely with broad domains delineated by the CpG islands of target genes [24] (Figure 1B). Furthermore, sequence-specific targeting via TFs is insufficient to account for localization of PcG proteins along the entire length of the inactive X chromosome (Xi) [25]. Xi targeting of PcG proteins has instead been attributed to direct interaction of PRC2 with the noncoding RNA (ncRNA) Xist [26], and several studies have since suggested a wider role for ncRNAs in PcG recruitment. At this time, the precise molecular mechanisms that underpin these proposed targeting mechanisms remain poorly defined (reviewed in [27]).

**Figure 2:**
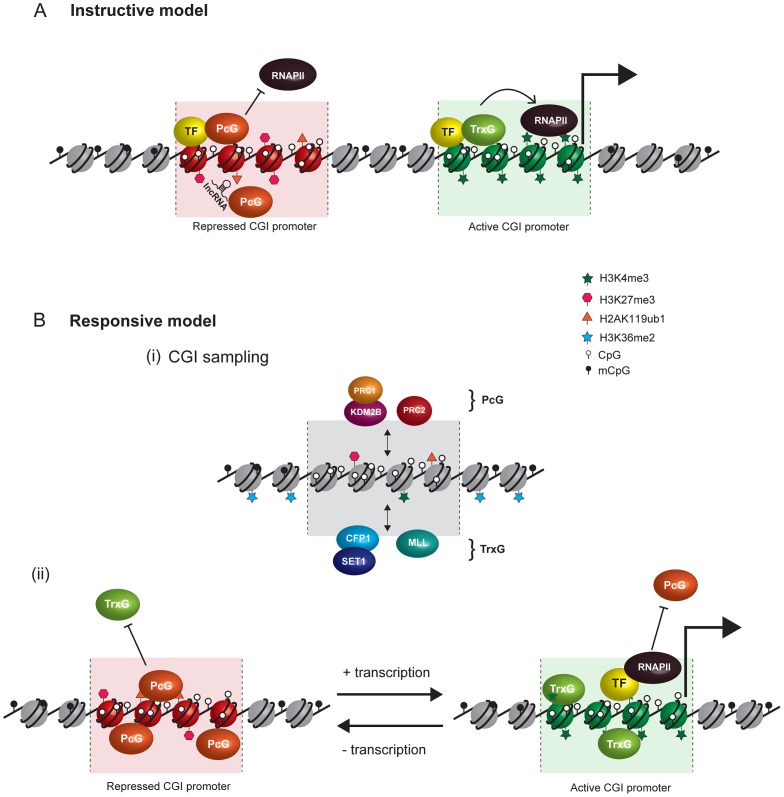
Comparison of instructive and responsive models for recruitment of PcG complexes to CpG islands at target gene promoters. DNA methylation and hypomethylation are indicated with filled and open lollipops, respectively. Repressed PcG–associated CpG islands are colored red and active TrxG–associated CpG islands green. (A) Classical instructive models invoke that sequence-specific DNA binding transcription factors (TF) and/or long noncoding RNAs (lncRNA) interact biochemically with PcG complexes and thereby target these complexes to defined promoters at which PcG-mediated histone modifications inhibit RNA polymerase II (RNAPII) activity. At active gene promoters (large arrow), TFs directly recruit TrxG proteins that in turn deposit histone modifications linked to gene activation. (B) In the responsive model, it is proposed that both PcG (PRC1-KDM2B and PRC2) and TrxG (CFP-SET1 and MLL) complexes stochastically sample unmethylated CpG island chromatin irrespective of the transcriptional state of a given gene (i). This occurs either via CxxC zinc finger protein binding to unmethylated CpG or, in the case of PRC2, via sensing the absence of otherwise pervasive histone modifications, like H3K36me2. The outcome of PcG and TrxG sampling would then be responsive to the transcriptional state of the associated gene (ii). In the absence of transcription, the PcG protein–occupied chromatin state would accumulate by default (aided by positive feedback loops) and antagonize TrxG activity. Conversely, at transcribed genes, TFs, RNAPolII, and transcription would favor accumulation of TrxG factors with their associated activities, in turn antagonizing the function of PcG complexes. Importantly, in the responsive model, the capacity of PcG proteins to sample CpG island chromatin would permit them to respond to the transcriptional state of potential target genes without a requirement for direct interactions with TFs or ncRNAs.

Current models invoking PcG recruitment through direct biochemical interaction with sequence-specific TFs and ncRNAs are summarized in Figure 2A. A central challenge with the current conceptual framework is to explain how PcG complexes are capable of interacting with the diversity of TFs and ncRNAs that would be necessary to account for the dynamic and lineage-specific PcG localization patterns that occur through development. This issue has been further exacerbated by the recent observation in mammals that noncanonical PRC1 complexes occupy their normal target gene promoters in different cell types and localize to the inactive X chromosome independently of PRC2 and H3K27me3, albeit at reduced levels [10]. As such, for TFs and ncRNA to fulfil the role as direct recruitment factors it would be necessary for PRC2 and variant PRC1 complexes to have independently evolved the capability to bind to the same diverse complement of targeting molecules.

## A Responsive Model for Targeting PcG Complexes

In light of accumulating challenges to the concept of instructive targeting of PcG complexes, we propose an alternative model based not on direct recruitment by sequence-specific TFs and ncRNAs, but rather on the recognition by PcG complexes of common chromatin features at target loci. This simplified and generic targeting regime would permit the concerted establishment of PcG repression at a subset of inactive loci, while counteracting features elsewhere would limit PcG protein activity. Within the confines of this conceptual framework, we envisage PcG activity at target loci will respond to the transcriptional state of the associated gene, as opposed to the prevailing view which posits that PcG protein function instructs transcriptional outcomes via physical interaction with and targeting by sequence-specific binding factors.

Key features of our model, focusing on PcG recruitment at target gene promoters, are illustrated in Figure 2B. The model is guided by the observation that PcG occupancy in vertebrate cells maps precisely to unmethylated CpG islands (CGI) at target gene promoters and the contention that these may function as the mammalian equivalent to PRE [24]. CGIs are short (1 to 2 kilobase) contiguous regions of DNA that escape the pervasive CpG dinucleotide DNA methylation characteristic of vertebrate genomes. Outside of CpG islands, DNA methylation represses transcription and contributes to repressive chromatin states. In contrast, CGIs are generally permissive to transcription and found in chromatin that is considerably more accessible than surrounding regions of methylated DNA [28]–[30]. Recent studies suggest that this relies, at least in part, on the activity of a family of zinc finger-CxxC (ZF-CxxC) DNA binding domain–containing proteins which recognize nonmethylated CpG dinucleotides and recruit chromatin modifying activities [31], [32]. CGIs are associated with more than half of vertebrate gene promoters, of which a subset in any given tissue appear to be very specifically occupied by the PcG proteins [33]. In fitting with a role for CGIs in PcG protein occupancy, the extent of PcG protein binding at individual loci correlates precisely with unmethylated CpG density within the CGI, not with the gene promoter or TSS [24]. Furthermore, an elegant series of genome engineering studies suggest that CGI characteristics are sufficient to recruit PcG proteins to ectopic sites [34], [35]; and related to this, a number of studies have identified a reciprocal relationship between PcG occupancy and DNA methylation [36]–[38].

In support of a role for CGIs in PcG recruitment, a direct molecular link between unmethylated CGIs and PcG localization has recently emerged. KDM2B, a ubiquitously expressed ZF-CxxC domain–containing protein, forms a noncanonical PRC1 complex which can bind to nonmethylated CpG sites [39]–[41]. Recognition of nonmethylated DNA by KDM2B allows this variant PRC1 complex to associate with most CGIs, albeit at levels which are extremely low in comparison to the levels of PRC1 that are observed at the subset of PcG-repressed CGI targets [41]. We propose that dynamic association of KDM2B with CGIs provides a means for PRC1 to sample potential target sites. Accumulation of PRC1 at a limited number of sites could then occur as a consequence of PRC1 monitoring the chromatin/transcription state within this limited search space. This simple but elegant sampling module would afford the necessary flexibility to allow PRC1 to engage all potential target sites in the genome, but ultimately only establish high-level PcG protein occupancy and silencing in a given cell type at a subset of susceptible CGIs. Importantly, this model circumvents the necessity for direct targeting of PRC1 by cell type–specific TFs, yet still allows a disparate complement of CGI target genes to be selected in developmentally diverse lineages. Moreover, KDM2B-mediated PRC1 sampling would be restricted to CGIs as CpG dinucleotides are underrepresented elsewhere in the genome and are pervasively methylated.

Could a similar CGI sampling activity also play a role in localization of PRC2 complexes? At present, there is no evidence for PRC2 binding preferentially to unmethylated CpG dinucleotides, but Jarid2, a substoichiometric PRC2 component, has been reported to bind GC-rich DNA sequences [42], . Alternatively, PRC2 selectivity for certain CGI targets might simply be achieved by its inherent preference for specific chromatin configurations and the limited capacity of its methyltransferase activity to modify histone tails with certain cis modifications [44], [45]. In support of this possibility, PRC2 activity is drastically inhibited by TrxG-mediated H3K4me3, a modification highly enriched at actively transcribed genes. Interestingly, the mammalian TrxG H3K4me3 methyltransferase complexes include proteins that contain ZF-CxxC domains, suggesting a dynamic CGI sampling mechanism could also guide TrxG and H3K4me3 to CGI promoters. PRC2 activity is also inhibited by a second modification, H3K36me2/me3. H3K6me2 is notable in this context as it is an abundant modification found at high levels throughout the genome but depleted at CGIs as a consequence of H3K36me1/2 demethylase activity of the ZF-CxxC domain proteins KDM2A and KDM2B [32], [46]. Thus, cis-inhibitory effects of these, and possibly other histone H3 tail modifications, may serve to define a chromatin state which is permissive to PRC2 occupancy and modification.

The idea that a dynamic sampling process may underpin the functionality of PcG complexes on chromatin is supported by the observation that PRC2 redistributes in mammalian cell lines [36]–[38], [47], [48] and in higher plant cells [49], [50] when DNA methylation is depleted. Similarly, in germline cells of *C. elegans* embryos, mutations in the MES4 protein, the major methyltransferase catalyzing H3K36me2, result in redistribution of PRC2 from the X chromosome where it is normally enriched to sites throughout the autosomes [51]. In both of these examples, loss of chromatin modifications that normally function to inhibit PcG activity in bulk chromatin appears to allow PcG activity to migrate to new sites, effectively titrating it away from bona fide PcG target sites.

## Feedback Mechanisms Stabilizing PcG and TrxG Chromatin States

If CGI hypomethylation, and linked to this, CGI-specific chromatin configurations, define sites where variant PRC1 and PRC2 can engage productively, what are the mechanisms that restrict H3K27me3 and H2AK119ub1 accumulation to nonexpressed targets? As outlined above, we propose that in a given cell type the subset of CGI sites that ultimately become PcG targets transition from a “sampled” state into an “established” PcG protein occupied and repressed state through the concerted action of both PRC1 and PRC2. This may be mediated through positive feedback loops driven by the function of their chromatin modifying activities. For example, PRC2-mediated H3K27me3 can lead to hierarchical recruitment of canonical PRC1 complexes via CBX proteins which bind H3K27me3. Furthermore, recently it was shown that PRC2 prefers to methylate compact chromatin substrates [44], a feature that PRC1 may potentiate [9].

Conversely, the majority of CGIs that remain free of established PcG silencing must possess a degree of “anti-silencing” that counteracts PcG protein establishment. How might this be achieved? One simple explanation would be that the major activity underpinning anti-silencing at most CGI promoters is the occupancy of positively acting transcription factors, the transcriptional machinery itself, and the function of TrxG proteins at these sites. Based on our evolving molecular understanding of PcG protein activity, there may be some sense in this idea. For example, although there exists some inherent level of H3K4me3 at CpG islands through ZF-CxxC domain–mediated targeting mechanisms [31], productive transcription dramatically amplifies this through RNA polymerase II–dependent recruitment of additional H3K4 methyltransferase activity [52]–[54]. As indicated above, PRC2-mediated H3K27 methylation is directly inhibited when the histone H3 tail carries H3K4me3. During activated gene expression, CGI regions also become extensively histone acetylated on H3 at position 27 (H3K27ac) which could have the added effect of directly blocking PRC2 activity and H3K27me3 [55]. Fittingly, it was recently shown that PRC2 fails to methylate H3K27 at some established PcG sites in cells lacking the NURD deacetylase complex which deacetylates H3K27ac [56]. Interestingly, several mammalian H3K4 methyltransferase complexes also associate with H3K27 demethylases, indicating a concerted effort to counteract PcG activity at CGIs during the process of H3K4me3 deposition and transcription [57].

If PcG proteins simply function to identify CGI loci that already lack significant transcriptional activity, in a manner that is responsive to transcription, why would their silencing services be required at all? One possibility lies in the fact that PcG systems appear to play important roles in keeping genes off in tissues where they should not normally be expressed. During development, as a cell transitions from one lineage to another, the expression of a subset of genes which are specific to the parental cell type must diminish in expression. A constant regime of CGI sampling would provide an opportunity for PcG proteins to identify these CGI sites in the genome, presumably by their lack of activated transcription and reduced capacity to anti-silence. Full and productive engagement of both PRC1 and PRC2 at these sites could then initiate, amplify, and fully establish a classical polycomb-repressed domain in response to this diminished anti-silencing. This would allow the cell to partition genes that have lost the capacity to be efficiently transcribed, an outcome that would be beneficial as it would help to protect the transcriptional identity of the cell from stochastic gene expression events that could lead to aberrant functionality. Furthermore, it would force the cell to invoke strong and persistent transcriptional signals to activate genes necessary for the transition to new and alternative transcriptional states. Not inconsistent with these ideas, removal of PcG proteins in embryonic stem cells affects transcription levels of only a proportion of target loci and then only by a small increment [24], [58]–[60]. We would suggest that this limited activation occurs as a result of basal activating signals present within the cell that are not of sufficient magnitude to overcome normal PcG-mediated silencing barriers.

The model we propose for PcG protein function in vertebrates is mechanistically rooted in an underlying requirement for nonmethylated DNA at CGIs, a feature that is not conserved in many invertebrate species which generally lack pervasive genome DNA methylation. Nevertheless, we believe that conceptually the interplay between “silencing” and “anti-silencing” at CGIs very much parallels the observations and ideas about PcG protein function that have emerged through the genetic and molecular study of the PcG system in *Drosophila*. For example, the type of positively acting chromatin modifying activities (remodelling ATPases, H3K4 and H3K36 methyltransferases) that we suggest are amplified with transcription and mechanistically correspond to anti-silencing at CGIs were originally identified in genetic interaction screens in *Drosophila* as PcG-counteracting TrxG proteins [14]. The direct mechanisms that lead to PcG and TrxG protein targeting to PREs in *Drosophila* still remain incompletely described. However, in light of our model, it is tempting to speculate that alternative sampling processes which do not rely on nonmethylated DNA may be at play in selecting PcG protein occupied sites in *Drosophila*. Indeed, there is indirect evidence that supports the possibility that PREs are continually sampled for susceptibility to PcG establishment [4], [61]. Moreover, the historical view that these outcomes should rely on TF-mediated interactions are not inconsistent with our model, but instead we would suggest these relationships may be less direct in nature with the system responding to the activity of TFs at PREs as opposed to directly mediating their outcomes.

## Broader Implications for the Role of Chromatin Structure in Transcriptional Regulation

**Figure 3:**
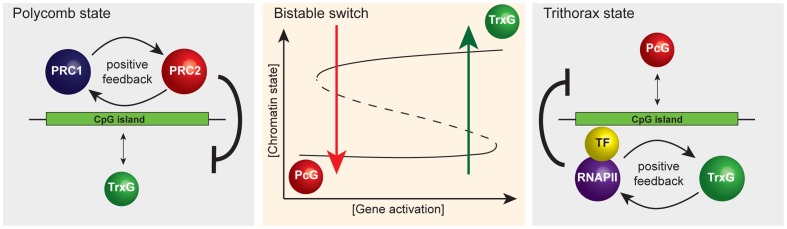
A simplified representation of the responsive model emphasizing that it has properties of a classical bistable switch. A schematic of the proposed bistable switch (center panel). The ground state at CpG island chromatin, PcG occupancy (left panel), is the default that is overcome only when TF-mediated gene activation reaches a critical threshold. Positive feedback mechanisms between PRC1 and PRC2 reinforce PcG occupancy and antagonize the TrxG state. Beyond the activation threshold, the system switches to the TrxG state (right panel) that is in turn reinforced by positive feedback mechanisms between RNAPII and TrxG factors. This, together with TrxG-mediated antagonism of PcG activities, ensures stability of the TrxG state such that stochastic fluctuations in gene activation signal would not trigger a switch back to the PcG state. However, when gene activation signals drop below a critical threshold, the CpG island will switch back to the default PcG state.

The concept of CGIs as sampling platforms for chromatin modifying enzymes has broader implications for how we view chromatin structure as part of transcription. If as we propose PcG and TrxG proteins respond, as opposed to instruct transcription, these factors would likely act as important modulators of transcriptional outcomes as opposed to drivers. This may be achieved by the creation of a bistable chromatin switch at CGIs through the opposing activities of PcG and TrxG complexes. For example, if PcG proteins can sample CGIs and then identify those that are in the nontranscribed state, positive feedback mechanisms linking PRC1 and PRC2 could help to reinforce a monostable chromatin state that is inhibitory to transcription (Figure 3). If CGIs are constantly sampled by both PcG and TrxG chromatin modifying activities, an opportunity to switch the chromatin state may constantly exist. For example, targeting of a strong and concerted activating signal to a PcG-occupied CGI promoter followed by productive transcription could permit exit from the PcG protein–repressed chromatin state coupled with transcription-associated deposition of H3K4me3. Several mammalian TrxG proteins encode domains that recognize H3K4me3, including the H3K4 methyltransferases themselves, perhaps leading to a transcription and TrxG-based positive feedback loop that defines a second transcriptionally permissive monostable chromatin state. Given that TrxG complexes in mammals contain activities that directly oppose PcG protein repressive function, this active chromatin state may play a modulatory role in sustaining transcription. Following cessation of transcription, the TrxG reinforcement and positive feedback loop sustained by this activity may correspondingly diminish. This transcription/TrxG anti-silencing effect could eventually be insufficient to counteract the PcG inhibitory state which may be reacquired by default. The net effect of this type of modulatory system presumably would be to ensure that the appropriate type and strength of activating signal is present before a gene is turned on and that the transcribed state can be sustained as long as the appropriate activating signal is present. Furthermore, individual chromatin states maintained by feedback mechanisms could contribute to mitotic heritability as defined in classical genetic experiments on PcG and TrxG systems.

Interestingly, high-resolution mapping of histone modifications in mammalian cells has revealed a class of repressed PcG CGI promoters at which TrxG-mediated H3K4me3, histone acetylation, and RNA polymerase II occupancy are all detectable, albeit at relatively low levels, referred to as bivalent promoters [62]–[64]. In the context of our model, we interpret that bivalency could represent the outcome of stochastic sampling of PcG-established CGIs by TrxG ZF-CxxC proteins or a degree of activator signal which is insufficient to switch the chromatin state. It will be interesting to examine in more detail the phenomena of bivalency in the context of a potential bistable chromatin state.

In support of the concept of chromatin bistability at CGIs, recent studies examining PcG chromatin modifications, TrxG chromatin modifications, and gene expression during stem cell differentiation also propose a bistable chromatin state that appears to describe the system with some degree of accuracy [65], [66]. Importantly, these studies suggest that local CpG density contributed by CGIs would be an important feature of such a system. Furthermore, modelling of chromatin dynamics and transcription during the process of plant vernalization, which utilizes polycomb systems, has also proposed that polycomb functions as part of a bistable chromatin switch [67]. Clearly, it will be interesting within the context of these concepts to design kinetic experiments to test the validity of such models in mammals and formally examine whether chromatin modulates transcriptional outputs consistent with the properties attributed to bistable systems.

## Concluding Comments

In summary, we suggest that our responsive model for PcG protein function in vertebrates overcomes many of the issues that complicate the prevailing instructive models, notably the requirement to invoke that PcG complexes physically interact with a unique set of TFs or ncRNAs in individual cell types. It should however be noted that the two types of model are not necessarily mutually exclusive. Furthermore, many of the features of our model adhere to the general principles describing PcG and TrxG protein function that were developed around observations from early genetic studies which suggest that these systems do not define transcriptional states but instead function to maintain predetermined transcriptional states [68]. In this respect, we believe the core molecular attributes of our model will provide a useful conceptual framework on which to experimentally examine its predictions and begin to better understand how the PcG and TrxG proteins function in gene regulation. Furthermore, it highlights the possibility that CGIs as core components of most vertebrate gene promoters may play a central role in modulating gene expression by providing a gene regulatory platform that is capable of contributing to a bistable chromatin environment at gene promoters. Although our description deals primarily with PcG recruitment at target gene CGIs in mammalian cells, we consider that the mechanisms invoked may be universal and at some level contribute to PcG recruitment at sites such as Xi, and also in other model systems, for example *C. elegans*, higher plants, and *Drosophila*.

## Note Added in Proof

A review of bivalent gene promoters that reaches similar conclusions to some of those expressed in this viewpoint was published when this article was under revision [69].

## Acknowledgments

Formulation of our model required the synthesis of many ideas and observations suggested previously by others and we wish to unequivocally acknowledge the derivative nature of our proposal. Due to limitations in the scope and depth of our viewpoint, we may have failed to reference all relevant papers and we apologize for any instances where this has occurred. We wish to thank Tom Milne, our lab colleagues, and the wider community for stimulating discussions.
